# Unilateral Glenoid Hypoplasia: A Case Report and Review of the Literature

**DOI:** 10.1155/2011/412721

**Published:** 2012-01-18

**Authors:** Ashish Suryawanshi, Amber Mittal, Snehal Dongre, Neeti Kashyap

**Affiliations:** ^1^Department of Orthopaedics, 6th Floor, MSB, Seth G. S. Medical College and KEM Hospital, Parel, Mumbai 400012, India; ^2^Department of Radiodiagnosis, Seth G. S. Medical College and KEM Hospital, Parel, Mumbai 400012, India; ^3^Department of Radiodiagnosis, B. J. Medical College, Pune 411001, India

## Abstract

Glenoid hypoplasia is a relatively rare alteration that in most cases involves the pectoral girdle in a bilateral and symmetrical manner. In general, glenoid hypoplasia is associated with skeletal changes such as hypoplasia of the humeral head or changes in the morphology of the acromion and of the coracoid. We describe a rare case of unilateral glenoid hypoplasia without instability and not involving humeral head. The patient was managed effectively with nonoperative measures that featured specific rehabilitation exercises for the shoulder.

## 1. Introduction

The term congenital and primary glenoid hypoplasia refers to an uncommon condition characterized by incomplete ossification of the lower two-thirds of the cartilaginous glenoid and adjacent neck of the scapula [1]. Since it was first described by Giongo [2] and Heupke [3], it has been reported in fewer than 100 patients.

 To our knowledge, in the largest series so far by Wirth et al, three groups of patients were identified: (1) those who had bilateral glenoid hypoplasia without instability of the shoulder, (2) those who had bilateral glenoid hypoplasia with instability of the shoulder, and (3) those who had unilateral glenoid hypoplasia and severe deformity of the humeral head [4].

 The purpose of this report is to describe a rare case of unilateral glenoid hypoplasia without instability of the shoulder and not involving the humeral head. The patient was managed effectively with nonoperative measures that featured specific rehabilitation exercises for the shoulder.

## 2. Case Report

A twenty-one-year-old male barber, right hand dominant, was seen at our hospital with six-year history of discomfort and mild pain in the left shoulder that had occurred with repeated overhead activity related to his occupation. No significant birth, family, medical, or surgical history could be obtained.

Physical examination revealed limitation of overhead abduction without any instability of the shoulder. Forward flexion was to 130 degrees on the left and to 140 degrees on the right, abduction to 80 degrees on the left and to 90 degrees on the right. Strength and tone examination revealed normal muscle strength. Muscle atrophy was absent and the examination was negative for ligamentous laxity. Laxity and stability examination revealed anterior apprehension to be negative, relocation test was negative, load and shift test was normal, the inferior sulcus sign was normal, and posterior load shift was normal as was adduction load. No generalized ligament laxity like hyperextension of metacarpophalangeal, knee, and elbow joint could be found.

**Figure 1:**
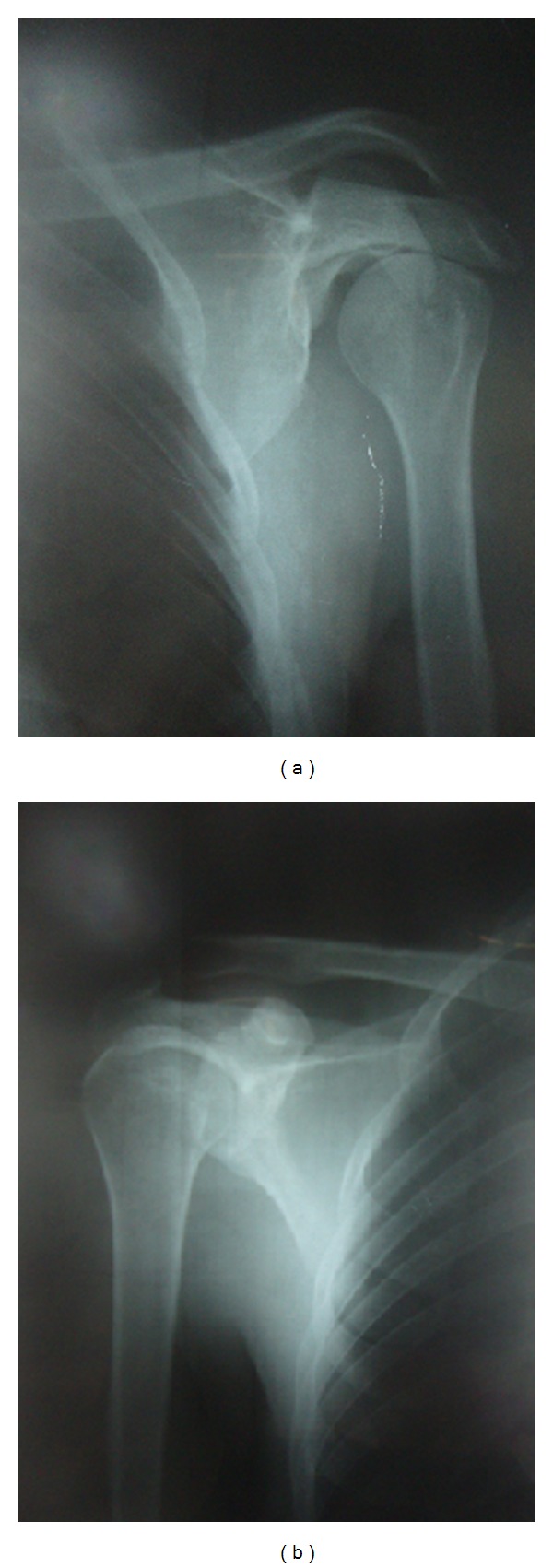
(a) shows Left-sided glenoid hypoplasia with normal humeral head. (b) shows normal right-sided shoulder radiograph.

**Figure 2:**
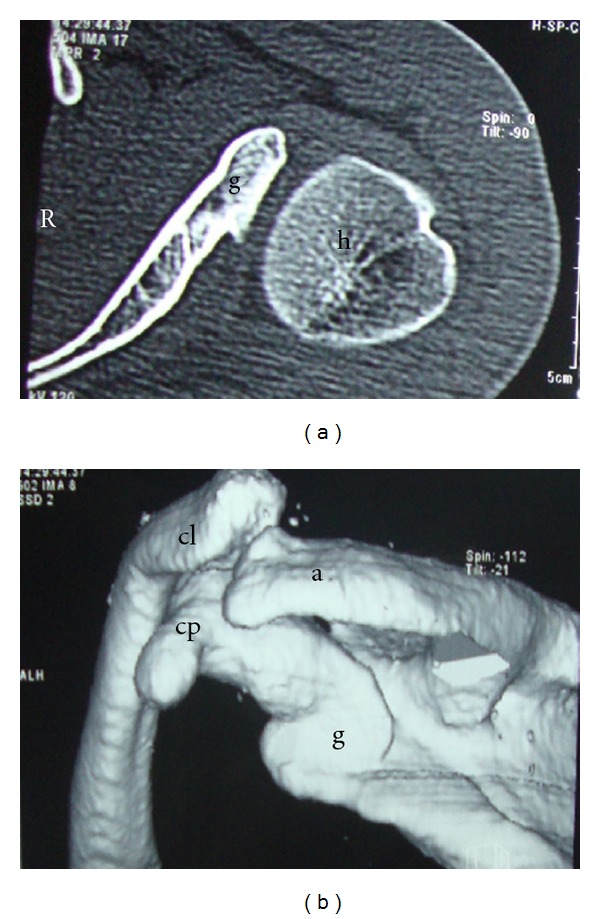
(a) CT scan showing (g) hypoplastic glenoid and (h) normal humeral head. (b) Three-dimensional CT scan showing (g) hypoplastic glenoid, (cp) prominent coracoid process, (cl) hooking of distal aspect of clavicle, and (a) prominent acromion.

Imaging studies (X-rays and CT scan) revealed hypoplastic glenoid fossa, prominent coracoid process, large acromion process, hooking of distal aspect of clavicle, and normal humeral head (Figures 1(a), 2(a), and 2(b)).

Investigations including levels of creatine kinase, vitamin C, vitamin D, liver, and renal function tests, EMG-NCV, were found to be normal.

Contralateral shoulder was found to be normal on history, physical examination, and imaging studies (Figure 1(b)).

The patient was managed conservatively using anti-inflammatory medications, moist heat application, life style modifications, rehabilitation exercises like rubber therabands, pulley kit and weight exercise, shoulder shrugs and push-ups (wall, knee and regular) to strengthen deltoid, internal rotators, external rotators, and scapular stabilising muscles and he fared well.

## 3. Discussion

The aetiology and inheritance of glenoid hypoplasia are poorly understood. The pathogenesis appears to be a failure of ossification of the inferior glenoid cartilage [1]. The underlying cause of this failure of ossification is not established. Before the report of Gardner and Gray' [5] from 1953 on the prenatal development of the human shoulder, little had been written about the embryology and histology of the shoulder. Developmentally, most of the scapula is formed by intramembranous ossification from eight centers or more. At birth, a large portion of the scapula is ossified, but the acromion, coracoid process, glenoid, vertebral border, and inferior angle of the scapula are cartilaginous. The glenoid fossa develops from a proximal and a distal center of ossification. The proximal, or subcoracoid, center appears around the tenth year and fuses at about the fifteenth year. At about this time, the second, or distal, center appears as a horseshoe-shaped epiphysis. This center is characterized by a thinner central portion surrounded by a thicker peripheral rim. An aberration of one or both of these ossification centers has been implicated in the development of glenoid hypoplasia. There appears to be a familial pattern in a small number of cases [6–8], but in most patients there is no definite family history. A number of conditions may be associated with a similar deformity [7, 9] including Erb's palsy, muscular dystrophy, aseptic osteochondritis, avitaminosis D, avitaminosis C, haemophiliac arthropathy, and neonatal septic arthritis [7].

 Samilson [10] suggest that individuals will probably become symptomatic in the second or third decade of life. The reported frequency of pain, restriction of motion, and instability of the shoulder in patients who have glenoid hypoplasia has been variable [4]. A review of the literature revealed that approximately 21 percent of patients have had discomfort in the shoulder, 43 percent have had some limitation of motion, and less than 2 percent have had symptomatic instability. The clinical findings in patients with primary glenoid hypoplasia may vary from severe pain and disability to no symptoms at all. The radiographic findings associated with glenoid hypoplasia have been well-described in the literature and include a shallow and irregular glenoid fossa, a prominent coracoid process, a large and elongated ribs, and flattening of the humeral head.

 Glenoid hypoplasia is often diagnosed as an incidental finding on chest radiographs as many patients with this disorder are asymptomatic. Edelson reported the incidence of posterior glenoid dysplasia in over 11.000 cadaveric specimens studied. As many as 35% of the specimens had deficiencies in the posterior-inferior aspect of the glenoid [11]. Pettersson [7] found that there was an inverse relationship between the degree of deformity and the range of motion of the shoulder. Our patient had limited motion with unilateral glenoid hypoplasia; radiographs revealed no degenerative changes of the joint but the marked glenoid deformity, apparent joint incongruity, and large overhanging acromion probably contributed to limited motion from a mechanical standpoint.

 The treatment of glenoid hypoplasia is controversial, with most authors advocating conservative measures [4, 12]. If recognized early in association with instability, then a glenoid osteotomy may be warranted to restores the normal version of the glenoid. In elderly patients who present with this abnormality in association with glenohumeral arthritis, total shoulder replacement may be an option.

 Smith and Bunker [13] noted that 75% of their patients had hooking or bossing of the distal clavicle and 50% of their patients had a prominent coracoids process. However, none of their patients had any abnormalities of the humeral head or neck, but involvement was bilateral. In contrast, the patient described by Collins et al. [14] had flattening of his glenoid with a notch inferiorly and slight flattening of the humeral head. Two patients described by Munshi and Davidson [15] had marked hypoplasia of the posterior glenoid and scapular neck with associated humeral head hypoplasia. Resnick et al. [16] also documented 2 cases with bilateral glenoid hypoplasia with humeral head involvement.

 In a review of the literature, Glenoid hypoplasia is usually bilateral and symmetric with only eight cases described in whom the findings were unilateral [4, 15, 17]. However, these cases have either shoulder instability or humeral head involvement. In conclusion, to our knowledge, our patient represents the only reported case of unilateral glenoid hypoplasia without instability and humeral head involvement which had been managed conservatively.
